# pWCP is a widely distributed and highly conserved *Wolbachia* plasmid in *Culex pipiens* and *Culex quinquefasciatus* mosquitoes worldwide

**DOI:** 10.1038/s43705-023-00248-2

**Published:** 2023-04-28

**Authors:** Amani Ghousein, Jordan Tutagata, Hans Schrieke, Manuel Etienne, Victor Chaumeau, Sebastien Boyer, Nonito Pages, David Roiz, A. Murat Eren, Guillaume Cambray, Julie Reveillaud

**Affiliations:** 1 MIVEGEC, University of Montpellier, INRAE, CNRS, IRD, Montpellier, France; 2 Centre de Biologie Structurale (CBS), University of Montpellier, INSERM U1054, CNRS UMR5048 Montpellier, France; 3 Centre de Démoustication et de Recherches Entomologiques - Lutte Anti-Vectorielle (CEDRE - LAV), avenue Pasteur, 97201 Fort-de-France, Martinique, France; 4 Shoklo Malaria Research Unit, Mahidol-Oxford Research Unit, Faculty of Tropical Medicine, Mahidol University, Mae Sot, Thailand; 5 Centre for Tropical Medicine and Global Health, Nuffield Department of Medicine, University of Oxford, Oxford, UK; 6 Institut Pasteur du Cambodge, Medical Entomology Unit, Phnom Penh, Cambodia; 7 ASTRE, University of Montpellier, CIRAD, INRAE, Montpellier, France; 8 CIRAD, UMR ASTRE, Guadeloupe, France; 9 International Joint Laboratory ELDORADO, IRD/UNAM, Mérida, México; 10 Marine Biological Laboratory, Woods Hole, Massachusetts, MA USA; 11 Helmholtz Institute for Functional Marine Biodiversity at the University of Oldenburg, Oldenburg, Germany; 12 Diversité des Génomes et Interactions Microorganismes Insectes (DGIMI), University of Montpellier, INRAE UMR 1333 Montpellier, France

**Keywords:** Bacterial genetics, Symbiosis

## Abstract

Mosquitoes represent the most important pathogen vectors and are responsible for the spread of a wide variety of poorly treatable diseases. *Wolbachia* are obligate intracellular bacteria that are widely distributed among arthropods and collectively represents one of the most promising solutions for vector control. In particular, *Wolbachia* has been shown to limit the transmission of pathogens, and to dramatically affect the reproductive behavior of their host through its phage WO. While much research has focused on deciphering and exploring the biocontrol applications of these WO-related phenotypes, the extent and potential impact of the *Wolbachia* mobilome remain poorly appreciated. Notably, several *Wolbachia* plasmids, carrying WO-like genes and Insertion Sequences (IS), thus possibly interrelated to other genetic units of the endosymbiont, have been recently discovered. Here we investigated the diversity and biogeography of the first described plasmid of *Wolbachia* in *Culex pipiens* (pWCP) in several islands and continental countries around the world—including Cambodia, Guadeloupe, Martinique, Thailand, and Mexico—together with mosquito strains from colonies that evolved for 2 to 30 years in the laboratory. We used PCR and qPCR to determine the presence and copy number of pWCP in individual mosquitoes, and highly accurate Sanger sequencing to evaluate potential variations. Together with earlier observation, our results show that pWCP is omnipresent and strikingly conserved among *Wolbachia* populations within mosquitoes from distant geographies and environmental conditions. These data suggest a critical role for the plasmid in *Wolbachia* ecology and evolution, and the potential of a great tool for further genetic dissection and possible manipulation of this endosymbiont.

## Introduction

The widespread intracellular bacterium *Wolbachia* has been at the heart of mosquito biocontrol programs for decades and is now more than ever triggering a surge of interest due to recent discoveries broadly related to its mobile genetic elements (its mobilome). Remarkably, *Wolbachia* is capable of manipulating the reproduction of its host, thereby favoring its own—almost exclusively maternal—spreading. It has also been shown to provide strong protection against the transmission of viral pathogens by mosquitoes [1–3]. Together, these properties champion *Wolbachia* as one of the promising strategies for vector control worldwide.

The most common effect of host reproduction manipulation, Cytoplasmic Incompatibility (CI), was recently found to be associated with the *cifs* genes harbored by *Wolbachia* bacteriophage WO [4–8]. These genes are part of a so-called Eukaryotic Associated Module (EAM) that presumably aids phage particles to cope with both prokaryotic and eukaryotic cell membranes, as well as cytoplasmic and extracellular host environments [9, 10]. WO commonly appears as a temperate prophage integrated in the chromosome and while lytic events remain rarely observed [9, 11], it provides a central source of evolutionary innovation and adaptation for the restricted lifestyle of the obligate intracellular endosymbiont [10, 12, 13].

Being an obligate intracellular symbiont, focus on the mobilome of *Wolbachia* spp. has long been restricted to WO, until the discovery of the first *Wolbachia* plasmid—named pWCP for “plasmid of *Wolbachia* in *Culex pipiens”*—opened new perspectives [14]. pWCP was originally reported from *Culex pipiens pipiens* specimens from the Mediterranean basin, including Southeastern Europe (France, Turkey) and Northern Africa (Tunisia, Algeria). However, the actual distribution and potential variability of pWCP in *Culex* mosquitoes remain unknown. Most pWCP-born genes were initially found exclusively in *Wolbachia* from *Culex quinquefasciatus* (*w*Pip, [15] and/or to the phylogenetic supergroup B-*Wolbachia*). Very recently, several homologous genes were identified in other *Wolbachia* supergroups from *Aedes albopictus* mosquitoes and other insect species, as part of other novel plasmids [16]. Similar to pWCP, plasmids of *Wolbachia* endosymbiont *w*AlbA 1 and 2 (namely pWALBA1 and pWALBA2) include a *parA*-like partitioning gene and a *RelBE* toxin–antitoxin system, supporting the likely functional importance of these elements in a plasmid context. In addition to putative phage-like proteins in pWALBA1, the authors reported for the first time *cif* genes homologs in the reconstructed plasmid from two reanalyzed *Wolbachia* genomes (Insecta_WOLB1166 and *D. virgifera virgifera*) together with plasmid-like islands located next to WO prophage regions in *O. gibbosus spiders* [16]. These novel data substantiate the idea that interactions between *Wolbachia* mobile genetic elements could enhance the adaptation and innovation capabilities of these endosymbionts. This remains to be further investigated in different mosquito species.

In this context, the presence of pWCP in *Culex* species requires critical attention. Indeed, the *Culex pipiens* complex represents the most widespread mosquitoes around the world [17, 18]. It is comprised of the tropical species *Culex quinquefasciatus* and the temperate species *Culex pipiens*, itself divided into two subspecies *Cx. pipiens molestus* and *Cx. pipiens pipiens*. Concomitant to its wide distribution, *Culex* species are vectors of numerous pathogens, causing a variety of known diseases that include West Nile Virus (WNV), one of the most commonly transmitted mosquito disease in the United States [19], St. Louis Encephalitis Virus (SLEV), Japanese Encephalitis Virus (JEV), Rift Valley Fever (RVF) [20] and the emerging virus Usutu (USUV) [21]. The combination of ubiquitous distribution, vector competence and opportunistic feeding behavior provides *Culex* mosquitoes with a high capacity to transmit infectious diseases between animal and humans (*i.e*., zoonoses), which represents an important threat to human health [22]. In fact, a large percentage of all newly identified infectious diseases are zoonoses, some of which have the potential to cause global pandemics, as recently demonstrated by the novel coronavirus that causes COVID-19 [23, 24].

Here, we collected *Culex* spp. (*C. pipiens and C. quinquefasciatus)* specimens from several continents and islands around the world including Thailand, Cambodia, Martinique, Guadeloupe, and Mexico along with laboratory colonies that have evolved for 2 years (ca. 24 generations) for *Culex pipiens molestus* and 30 years (ca. 360 generations) for *Culex quinquefasciatus* SLAB in artificial conditions, and screened for the presence and variability of pWCP in the germline and somatic tissues of these widespread samples.

## Results

### Screening of pWCP in *Wolbachia*-infected *Culex* samples

**Fig. 1:**
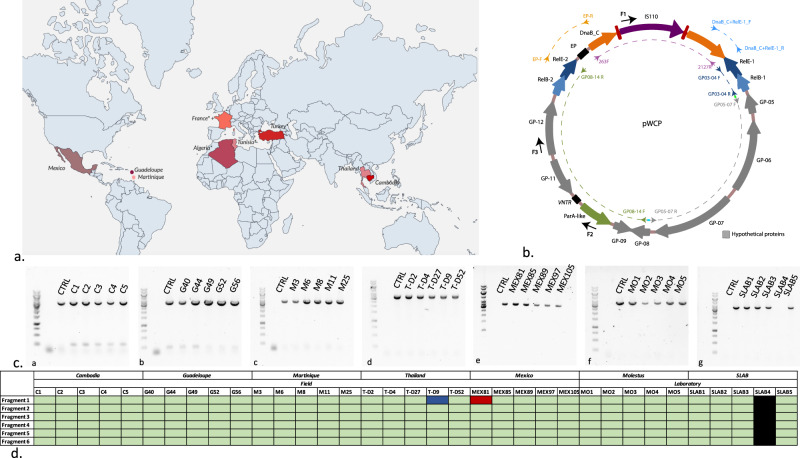
pWCP distribution and sequence variability. **a** Geographic map with locations of *Culex* sample collection for pWCP screening. Colors indicate field samples with different color for each country sampled. A “+” indicates laboratory specimen and “*” indicates previous observations from [14]. **b** Map of pWCP plasmid (adapted from [14]). Genes are shown as filled arrows. Couples of PCR primers spanning different regions of the plasmid designed to cover pWCP are shown as smaller arrows. Dash lines indicate amplified fragments (Purple: Fragment 1; Dark blue: Fragment 2; Gray: Fragment 3; Green: Fragment 4; Orange: Fragment 5; Light blue: Fragment 6). Bright green and blue dots indicate overlapping primers as highlighted in Supplementary Table 2. Additional sequencing primers are shown in black arrows on the outer layer. **c** PCR amplification of the largest and most representative for plasmid genetic diversity Fragment 4 in studied samples. A ca. 3451 bp PCR product corresponding to the amplification of Fragment 4 including seven genes of pWCP (GP08, GP09, ParA-like, VNTR, GP11, GP12 and RelBE-2) in most ovary samples collected from different regions. a: Cambodia, b: Guadeloupe, c: Martinique, d: Thailand, e: Mexico, d: Montpellier (molestus) and f: SLAB. The first raw of each gel is the negative CTRL. **d** Synthetic heatmap of conserved pWCP fragments across samples. pWCP fragments 1–6 are shown in row and studied samples in column. Green color indicates fragment of the right size, blue if longer, red if shorter. Black corresponds to *Wolbachia*-free sample.

We collected and dissected the ovaries and midguts of field *Culex quinquefasciatus* mosquito specimens from Cambodia, Thailand, Guadeloupe, Martinique, and Mexico (Fig. 1a, Supplementary Table 1). In addition, we sampled *Culex pipiens molestus* specimens originating from a colony that we collected in Montpellier (South of France) and maintained in the laboratory for 2 years (2020–2022) as well as a *Culex quinquefasciatus* SLAB samples, which have been kept more than 30 years in lab environment. Our sampling effort including 35 *Culex* specimens aimed to search for pWCP in mosquitoes from both continental and islands areas across the globe, as well as distinct environmental and laboratory settings.

We first confirmed the presence of *Wolbachia* in the ovaries of the different *Culex samples*. A PCR amplification using specific primers targeted to the 16 S ribosomal RNA gene of *Wolbachia* clearly showed that all collected *Culex* specimens were infected by *Wolbachia* (Supplementary Fig. 1, Supplementary Table 2), except for one of the five SLAB samples. Although a sensitivity issue due to very low *Wolbachia* density or degraded DNA could possibly explain the lack of PCR amplification for this ovary sample, it is also possible that *Wolbachia* was not transmitted in SLAB4 in this laboratory colony.

We then screened for the presence of pWCP in the 34 *Wolbachia*-positive *Culex* samples. For that purpose, we designed and used six sets of primers spanning overlapping regions of the plasmid to eventually cover the entirety of pWCP (Fig. 1b). All sets of primers produced amplicons of the expected size in all but 2 samples, T-D9 from Thailand and MEX81 from Mexico, which respectively produced larger and smaller amplicons for fragment 1 (ca. 3000 and 400 bp instead of 1800 bp) (Supplementary Figs. 2–6). These data indicated that no major recombination events had occurred with respect to the reference pWCP.

To further investigate whether the plasmid occurred in mosquito somatic tissues, we performed PCR screens on midgut samples isolated from the same *Wolbachia*-infected individuals (two per origin) for 3 of the fragments. All samples were positive for the presence of *Wolbachia*. We could only observe faint bands in 3 out of 14 samples for each pWCP fragment tested. This likely reflects a lower abundance of the plasmid in non-germinal organs (Supplementary Fig. 7) and may be related to lower *Wolbachia* densities generally observed in somatic tissues.

### Estimating pWCP variability in *Culex* spp. across the globe

We next sought to quantify the extent of sequence diversity present in the pWCP genome across different geographical regions and conditions. For this, we Sanger sequenced the PCR products obtained above in the 34 *Wolbachia*-infected *Culex* specimens and aligned the resulting sequences against our reference pWCP (see Supplementary Table 3 for sequencing primers). We observed no variation in Fragment 1 (925 nts, Alignment 1 A; 918 nts, Alignment 1B), Fragment 2 (533 nts, Alignment 2), Fragment 3 (712 nts, Alignment 3), Fragment 4 (970 nts, Alignment 4 A, except for the variable number tandem repeat (VNTR) region as expected and described in Alignment 4B below; 998 nts Alignment 4 C, and 901 nts Alignment 4D), nor Fragment 5 (613 nts, Alignment 5) and Fragment 6 (325 nts, Alignment 6) where each of the sequences from the set of *Culex* samples were 100% identical, revealing a high level of conservation across continents (Fig. 1d).

The exact sequence of the smaller Fragment 1 amplicon obtained in sample MEX81 can be obtained by deleting the sequence of IS110 *in silico*. This precise excision re-establishes the correct reading frame of the *DnaB-C* gene (Alignment 1 C). A BLASTP search for the translated sequence identifies a hit from an unclassified *Wolbachia* species with 97% identity and a perfect match around the excision point (WP_264337168.1), which further suggests that the excision re-establish a functional protein. In contrast, a BLASTP search using a 375 nts ORF identified in the longer Fragment 1 amplicon from sample T-D9 identified an IS630-related transposase in *Wolbachia* of *Culex quinquefasciatus* (e.g., WP_012481719.1, 97% identity over 118 residues), thus hinting at the probable insertion of another IS within the IS110 already inserted in our reference pWCP (Alignment 1D).

The variable number tandem repeat (VNTR) region of pWCP had previously been identified as a polymorphic locus (pp-hC1A_5) and used for typing different strains of *Wolbachia* [25]. We previously found it to be variable across individuals isolated in the Mediterranean region [14]. Here, we observed an insertion of 16 bp with respect to pp-hC1A_5 of the original pWCP reference sequence in all specimens but T-D2 and MEX85, as well as punctual mutations in all samples (see Alignment 4B), which further reveal the variable nature of the VNTR region at the individual level.

Overall, our data demonstrate an unexpectedly high degree of conservation of pWCP around the globe.

### pWCP copy number in *Culex* spp. worldwide

To obtain further insights on the behavior of the pWCP plasmid, we investigated its copy number in ovary samples collected from different localities and conditions (3 specimen per origin, Supplementary Table 4 sheet 2) using qPCR and a *Culex pipiens* specimen from Southern France as control (Cx1, from the same locality as specimen studied in [14]). Globally, we found an average pWCP copy number of ca. 9 with seemingly high variations across locations and individuals (standard deviation of ca. 12). Three quarters (76%) of the observed variance in copy number is explained by differences between locations, while the remaining quarter (24%) correspond to variation between individuals within locations. Closer inspection revealed that most of this variance (78%) is actually contributed by SLAB specimens from the colony that has been maintained in laboratory conditions for over 30 years, which show an elevated copy number of ca. 32. Excluding these specimens, the copy number of pWCP falls to ca. 5 ± 4, which is consistent with numbers previously derived from next generation sequencing of samples collected in the Mediterranean region [14]. The variance is then still dominated by copy number variations amongst locations (68%) rather than between individuals within locations (32%), but none of the differences between locations are statistically significant (see Supplementary Table 5 for statistical analyses and Supplementary Fig. 8). Our data thus point to a rather stable copy number worldwide, with inter-individuals changes that may reflect different physiological states of the individual mosquito specimens.

## Discussion

We screened for the presence and variability of pWCP among 30 *Culex quinquefasciatus* and 5 *Culex pipiens molestus* specimens, sampled across the European, North American and Asian continents as well as from several islands. Our collection included freshly collected wild specimens together with samples originating from mosquito strains maintained in the lab for two to more than 30 years. PCR and Sanger sequencing results indicate that pWCP is widely distributed and highly conserved among *Culex* spp. worldwide. We observed identical pWCP in nearly all *Culex* mosquito specimens studied, which strongly support the notion that the presence of pWCP is not incidental and that its ordered and highly conserved genes are likely to be functional. This is reminiscent of the remarkably high average nucleotide identity observed across *Wolbachia* genomes reconstructed from Southern France and the *w*Pip Pel reference genome originally isolated in Sri Lanka (99.1–99.98%) [14], which suggested a high degree of core and essential genome conservation across individuals.

We only found polymorphism associated with the VNTR region and the IS110 transposase. The VNTR region is a known polymorphic hotspot due to its high repeat content that favor polymerase slippage during replication [14, 25]. It is, therefore, not surprising that we found different repeat numbers and/or punctual mutations in all samples. However, the functional significance of these variations remains to be established. As in our reference pWCP, IS110 was found inserted within the *DnaB-C* gene in all but one sample (MEX81), where it has excised in frame to yield a likely functional helicase gene. This suggests that the insertion is an ancestral event and that the IS is still functional and can occasionally excise in a precise fashion. A complete non-functionalization of *DnaB-C* consecutive to the insertion would be expected to cause the accumulation of mutations in the corresponding gene. The lack of variation observed in this region within our samples suggests that the locus is in fact not a pseudogene, but could code for two separate *DnaB-C* subunits that could assemble into a functional complex. Of note, one sample from Thailand (D9) showed the presence of an extra sequence within IS110, which produces hits against IS630 of *w*Pip by BLASTP. These two latter ISs are frequently found in WO genomes [26], which further suggest that exchanges between the different types of *Wolbachia* mobile genetic elements are possible.

It is particularly striking that completely identical pWCP were found in both field and laboratory settings. These findings highlight that the circular element is maintained across generations in very different environments and associated selective pressures. In line with this, we observed the presence of pWCP in the ovaries as well as the midguts of *Culex* specimens, suggesting that pWCP follows the trajectories of *Wolbachia* transmission from germlines to somatic tissue.

Previous data obtained using next-generation sequencing from samples isolated in France, Turkey, Algeria, and Tunisia concluded at a low pWCP copy number comprised between 4 and 7 (Supplementary Note 1 in [14]). In this study, we confirm a rather low copy number estimated to 5 ± 4 across wild specimen collected worldwide using a different technical approach (qPCR). We found sizable variations between individuals, which could very well reflect the impact of distinct physiological states of the specimens. For example, Martinez and colleagues reported that copy numbers of the plasmid pWALBA2 tend to vary in function of age, with increased numbers in older female mosquitoes [16]. Interestingly, we observed a much higher pWCP copy number (ca. 32) in laboratory SLAB specimen. In the absence of systematic pWCP sequence variations associated with these sample, it is possible that this change results from a different physiology in this colony that has evolved for more than 30 years in husbandry conditions. Further studies would be needed to investigate this further.

Overall, the detection and conservation of pWCP in three species of the *Culex pipiens* mosquito complex (*Cx. quinquefasciatus, Cx. pipiens pipiens* and *Cx. pipiens molestus*) across four continents (North Africa [14], Europe, America and Asia) strengthen the notion that this mobile genetic element plays an important role in *Wolbachia* biology. These observations open perspectives for the development of a genetic engineering tool that could help unraveling the complex molecular mechanism of interactions between *Wolbachia*, its associated mobilome and the host in these and possibly other species. Such tool and derived knowledge could help devise novel vector control strategies that may have great impact in the battle against pathogens spread from diverse mosquito species [27].

## Materials and methods

### Mosquito sampling

Collection and dissection of mosquito specimens was performed as described in [14] following a standardized protocol for each location. Briefly, we collected mosquitoes using a carbon dioxide mosquito trap (BG-Sentinel with BG lure or CDC trap baited with carbon dioxide) or an aspiration device and transported them alive to the laboratory directly afterward. Females were identified at species-level, anesthetized by incubation at −20 °C for 4 min (min), surface-sterilized with ethanol 96% for 1 min, quickly rinsed with sterile PBS to avoid DNA fixation by ethanol, transferred in a drop of sterile PBS deposited on a sterile microscope slide and dissected using sterilized tweezers. Ovaries and midgut from each single mosquito were separated and stored in sterile buffer to preserve them until further processing.

### DNA extraction and PCR amplification

DNA from each organ was extracted using a Qiagen DNeasy blood and tissue kit according to manufacturer’s instructions after rinsing samples with 1000 µl PBS and centrifugation at 12,000 g at 15 °C for 15 min. DNA was quantified using Qubit (Supplementary Table 1). We used primer CQ11F2 (5’-GATCCTAGCAAGCGAGAAC-3’) and pipCQ11R (5’-CATGTTGAGCTTCGGTGAA-3’) and molCQ11R (5’-CCCTCCAGTAAGGTATCAAC-3’) to confirm the taxonomy of *Culex pipiens pipiens* versus *molestus* [27]. The presence of *Wolbachia* was monitored by amplifying the 16 S rRNA gene using Wspec F and R primers [28], while the presence of pWCP was investigated by using six sets of specific primers as follows: 263 F and 2127 R for fragment 1, GP03-04F and GP03-04R for fragment 2, GP05-07F and GP05-07R for fragment 3, GP08-14F and GP08-14R for fragment 4 and EP-F and EP-R for fragment 5, as well as DnaB_C + RelE-1_F and DnaB_C + RelE-1_R for fragment 6. All primer sequences are listed in Supplementary Table 2.

PCR amplifications were performed using 1 ng of DNA as template material, 5 μL of 5x reaction buffer, 1 μL dNTPs (10 mM), 0.25 μL of Phusion DNA polymerase (NEB) and nucleic-acid-free water to a final volume of 25 μL. PCR products were electrophoresed on a 0.8% agarose gel to determine the presence of the desired size product of the amplified DNA, using GeneRuler 1 kb (Thermo Scientific SM0313) as a ladder. DNA from an ovary *Culex pipiens pipiens* sample collected in Montpellier, France (hereafter Cx1) was used as positive control for PCRs and gels. A negative control devoid of template material was also run within each set of reaction.

### Sanger sequencing and blast searches

We Sanger-sequenced all PCR amplicons obtained from two randomly chosen samples from each geographic location and lab colonies and show one representative sample for each after checking for the lack of intra-site variation. PCR products were purified using Monarch PCR & DNA Cleanup Kit (NEB) for all fragments, except Fragment 1. Since the PCR for fragment 1 showed substantial non-specific bands, the band at ca. 1800 bp was excised and processed using Monarch DNA Gel Extraction kit (NEB). Purification kits were used according to manufacturer’s instructions. The purified PCR products were premixed with different corresponding primers and sent for sequencing using ‘name of the sequencing product’ (Eurofins). We used BLASTN (MegaBLAST) and BLASTP from the NCBI’s web servers to search for DNA and proteins sequences similar to Fragment 1 sequences for samples MEX81 and T-D9, using default parameters against the nr/nt and nr database, respectively.

### Plasmid copy number determination by quantitative real-time PCR

We performed real-time qPCR on three samples from each locality. *Culex pipiens Cx1* from Southern France was used as a control (CTRL). We performed the qPCR reactions in triplicates using 10 μL mixtures (5 μL of SYBR^TM^ Select Master Mix (Thermo Fisher Scientific; 4472897), 1 ng of DNA template, 600 ng of each primer, adjusting the total volume to 10 μl using nuclease-free water) for each reaction. We used two new primers designed after [14] to target GP10 of pWCP (GP10F and GP10R, Supplementary Table 4, Sheet 1) and wolpipdir and wolpiprev primers [29] to target *Wolbachia*’s gene *wsp*.

We established the plasmid DNA standard curve by performing serial dilutions of a synthesized plasmid (Eurofins) designed to include both *gp10* and *wsp* genes. We plotted the standard curves as the Ct values versus the log concentration of the standard plasmid DNA. For calculating the samples’ absolute quantity of the plasmid, we interpolated each Ct value against the standard curve for both used primer sets. We then calculated the plasmid copy number by dividing the copy number of *gp10* by the copy number *wsp*. Statistical analyses were performed using one-way Anova multiple comparisons test in GraphPad Prism (Supplementary Table 5).

## Supplementary Information

Supplementary_Material

Supplementary_Table1

Supplementary_Table2

Supplementary_Table3

Supplementary_Table4

Supplementary_Table5

## Data Availability

Sequencing data for pWCP is available at 10.5281/zenodo.7039954.
