# Supplementary material for: pWCP is a widely distributed and highly conserved Wolbachia plasmid in Culex pipiens and Culex quinquefasciatus mosquitoes worldwide
Source: ISME Commun. 2023 Apr 28;3:40. doi: 10.1038/s43705-023-00248-2 (PMC10144880; doi:10.1038/s43705-023-00248-2)
Supplement: Supplementary file 1 — Supplementary_Material [file 43705_2023_248_MOESM1_ESM.pdf]

Supplementary Material for "pWCP is a widely distributed and highly conserved *Wolbachia* plasmid in *Culex pipiens and Culex quinquefasciatus* mosquitoes worldwide"

Supplementary Table 1. Collected samples together with their associated metadata including individual number, dissected organ, storage buffer, location, field vs. lab settings, date of collection, feeding status, gravid status, person who realized dissection, DNA concentration.

Supplementary Table 2. Primers and conditions used for the PCR amplification of the six pWCP regions. Colored nucleotides (nt) indicate overlapping nt between fragments.

Supplementary Table 3. Primers used for the sequencing of the six pWCP regions and the corresponding alignments

Supplementary Table 4. Sheet 1 illustrates the primers that were used for qPCR to determine pWCP copy number. Sheet 2 shows the three specimens from each locality that were used to perform the qPCR to determine pWCP copy number.

Supplementary Table 5. Statistical analysis using one-way Anova multiple comparisons test to assess statistical significance of pWCP copy number difference at the inter (Sheet 1) , intra-locality level (Sheet 2) and decomposition of variance using all data (Sheet 3).

Alignments. Alignment 1A. Sanger sequencing of Fragment 1 for one sample per location using primer 2127R. **Alignment 1B.** Sequence of Fragment 1 for one sample per location using primer F1. **Alignment 1C**. Sequence of Fragment 1 for sample MEX81 using primer 2127R. **Alignment 1D.** Sequence of Fragment 1 for T-D9 using primer F1. **Alignment 2.** Sanger sequencing of Fragment 2 for one sample per location using primer GP03-04F. **Alignment 3.** Sanger sequencing of Fragment 3 for one sample per location using primer GP05-GP07R. **Alignment 4A.** Sanger sequencing of Fragment 4 for one sample per location using primer GP08-14F. **Alignment 4B**. Sanger sequencing of Fragment 4 for one sample per location using primer F2. **Alignment 4C.** Sanger sequencing of Fragment 4 for one sample per location using primer F3. **Alignment 4D.** Sanger sequencing of Fragment 4 for one sample per location using primer GP08-14R. **Alignment 5**. Sanger sequencing of Fragment 5 for one sample per location using primer EP-F. **Alignment 6**. Sanger sequencing of Fragment 6 for one sample per location using primer DnaB_C+RelE-1_F.

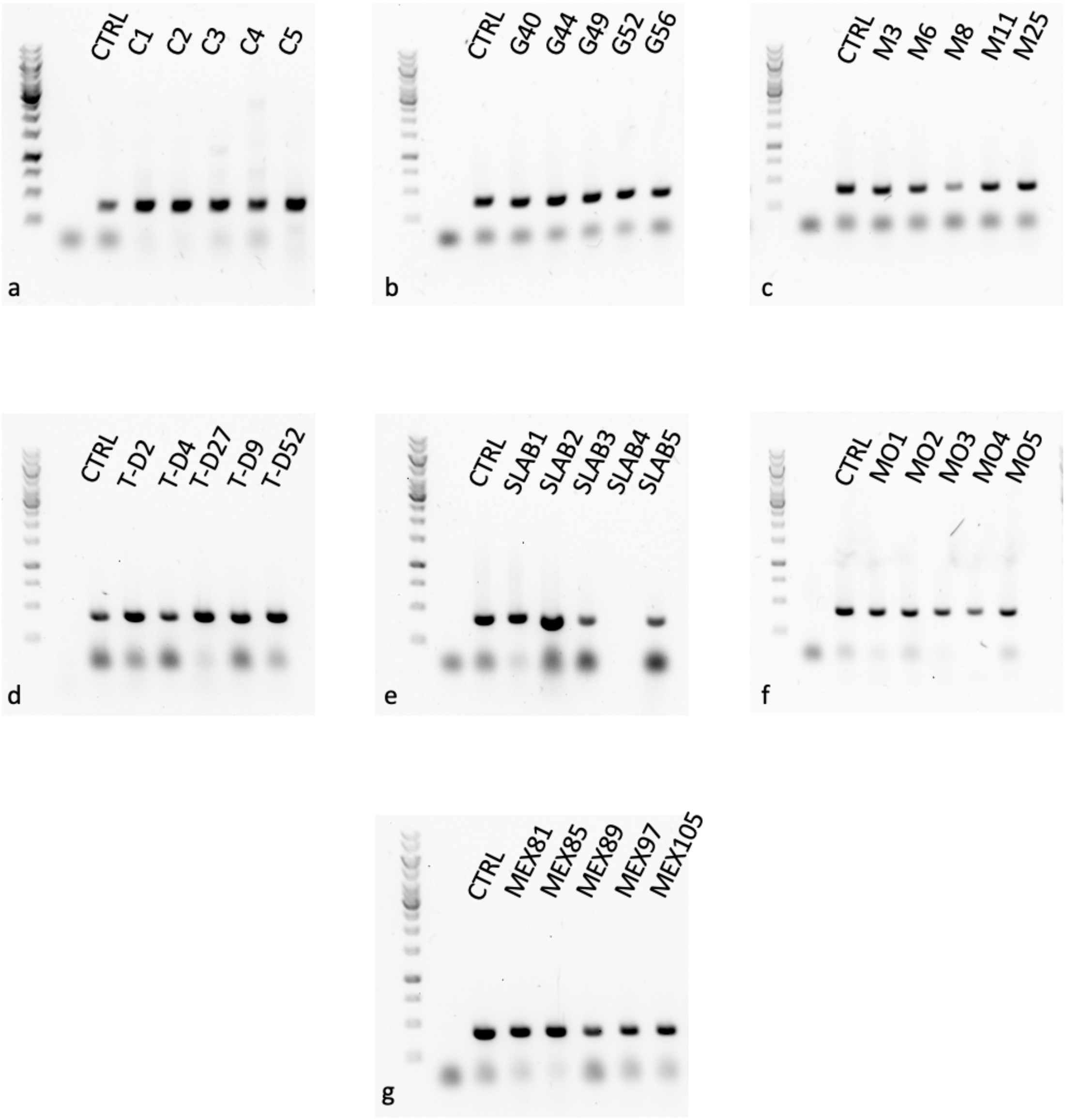

Supplementary Figure 1. A ca. 438 bp PCR product corresponding to the amplification of 16S rRNA gene in most ovary samples collected from different regions. a: Cambodia, b: Guadeloupe, c: Martinique, d: Thailand, e: SLAB, f: Montpellier (molestus) and g: Mexico. (Second lane on the gel corresponds to the negative CTRL sample).

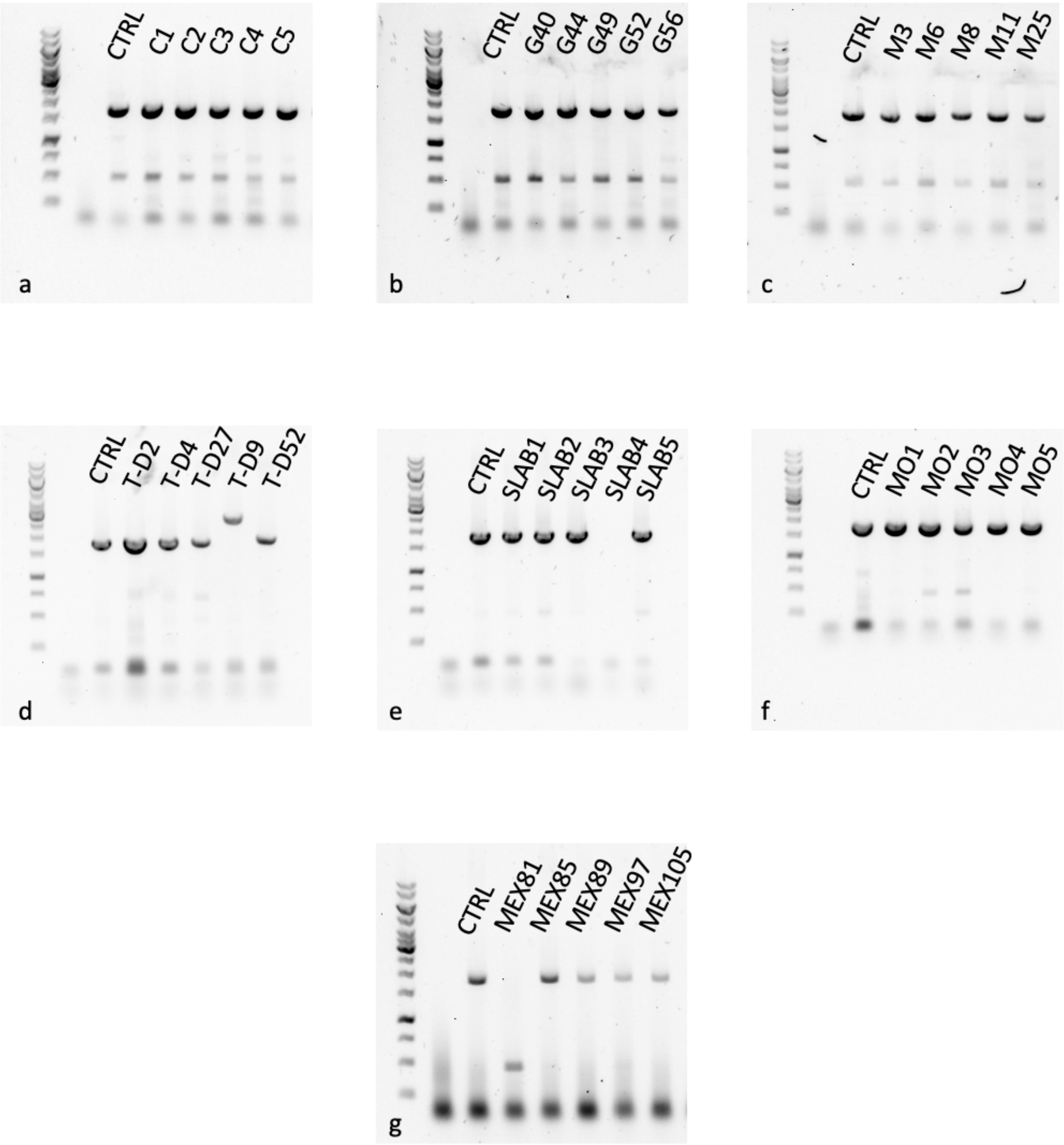

Supplementary Figure 2. A 1800 bp PCR product corresponding to the amplification of Fragment 1 of pWCP including *DnaB_C* gene and IS110 in most ovary samples collected from different regions. a: Cambodia, b: Guadeloupe, c: Martinique, d: Thailand, e: SLAB, f: Montpellier (molestus) and g: Mexico. The lower band in several samples of ca. 500 bp corresponds to the amplification of *DnaB_C* gene from *Wolbachia* genome as previously observed in [1]. (Second lane on the gel corresponds to the negative CTRL sample).

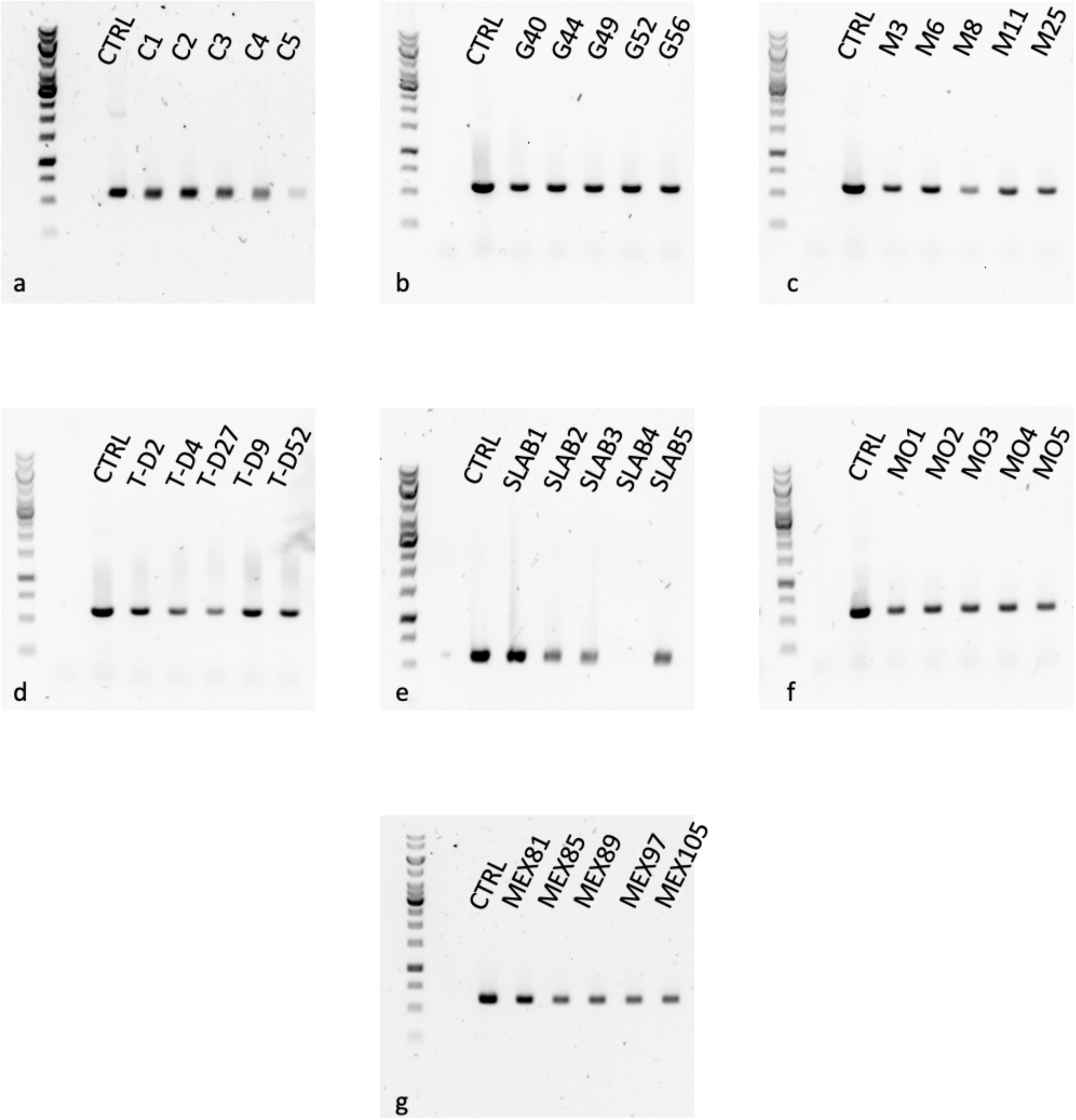

Supplementary Figure 3. Amplification of a 609 bp product (Fragment 2) covering GP03-GP04 of pWCP in most ovary samples collected from different regions. a: Cambodia, b: Guadeloupe, c: Martinique, d: Thailand, e: SLAB, f: Montpellier (molestus) and g: Mexico. (Second lane on the gel corresponds to the negative CTRL sample).

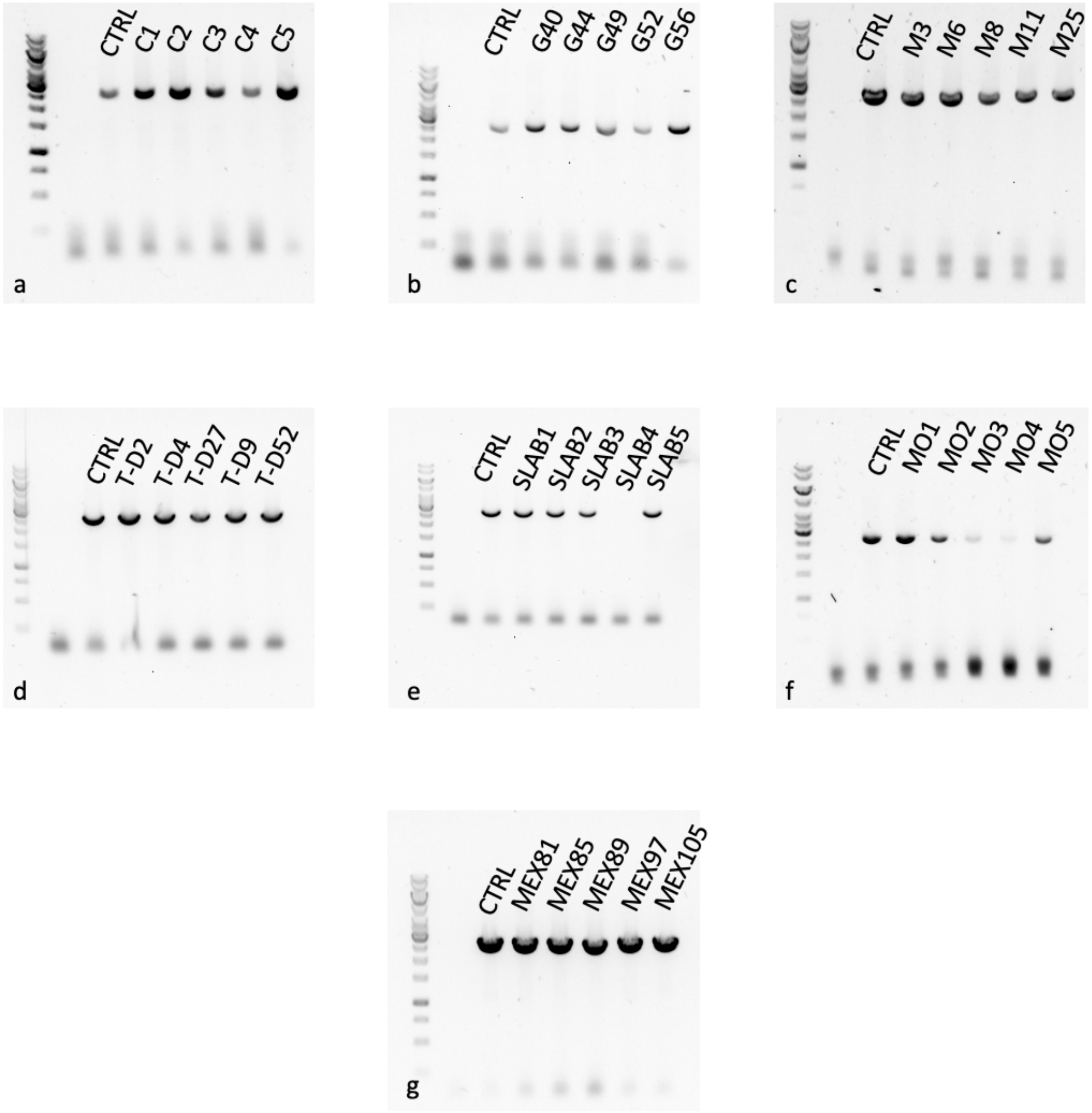

Supplementary Figure 4. A ca. 2726 bp PCR product corresponding to the amplification of Fragment 3 including GP05, GP06 and GP07 in most ovary samples collected from different regions. a: Cambodia, b: Guadeloupe, c: Martinique, d: Thailand, e: SLAB, f: Montpellier (molestus) and g: Mexico. (Second lane on the gel corresponds to the negative CTRL sample).

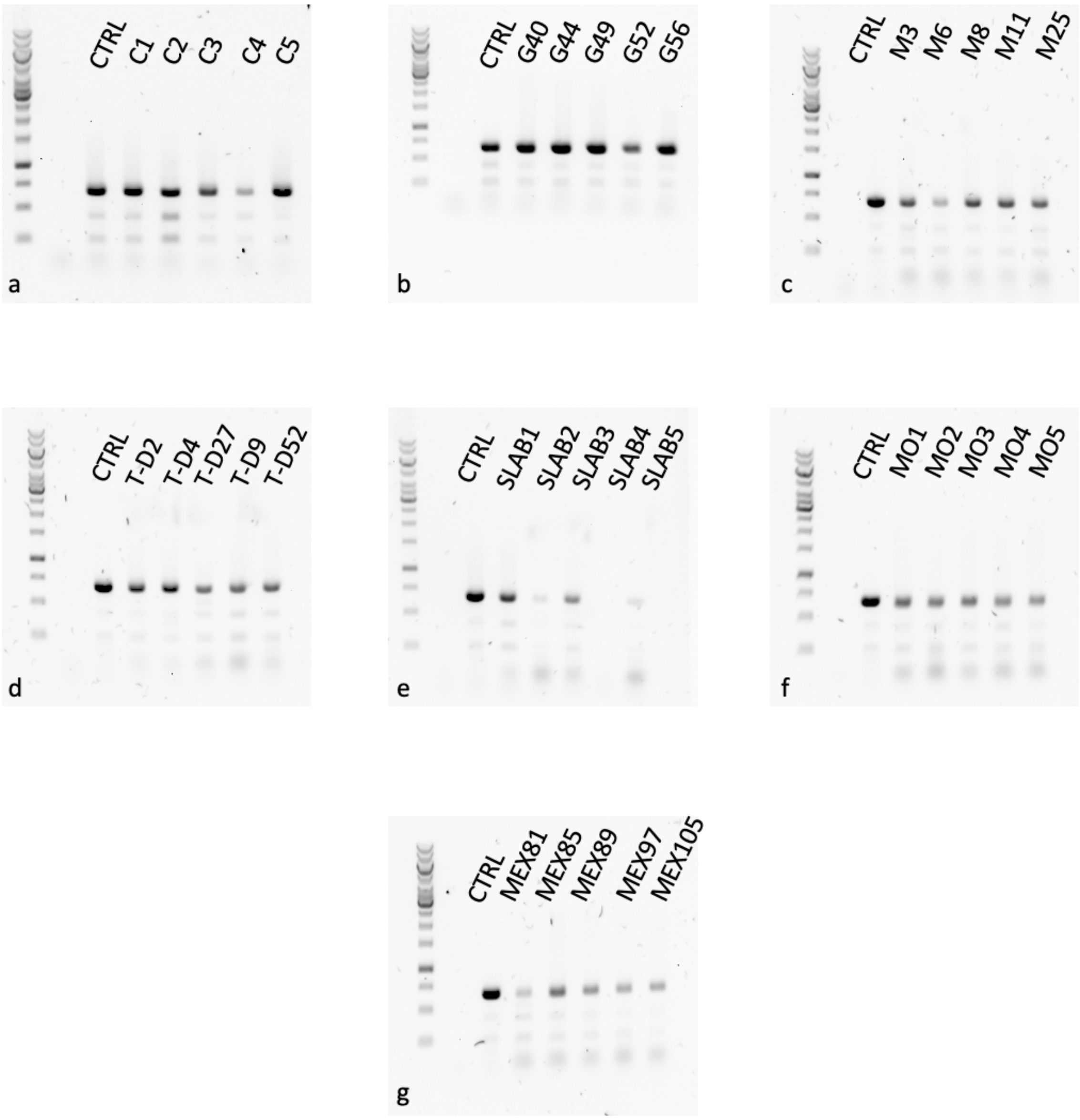

Supplementary Figure 5. A ca. 678 bp PCR product corresponding to the amplification of Fragment 5 including the *RelE-2*, EP and *DnaB_C* region of pWCP in most ovary samples collected from different regions. a: Cambodia, b: Guadeloupe, c: Martinique, d: Thailand, e: SLAB, f: Montpellier (molestus) and g: Mexico. (Second lane on the gel corresponds to the negative CTRL sample).

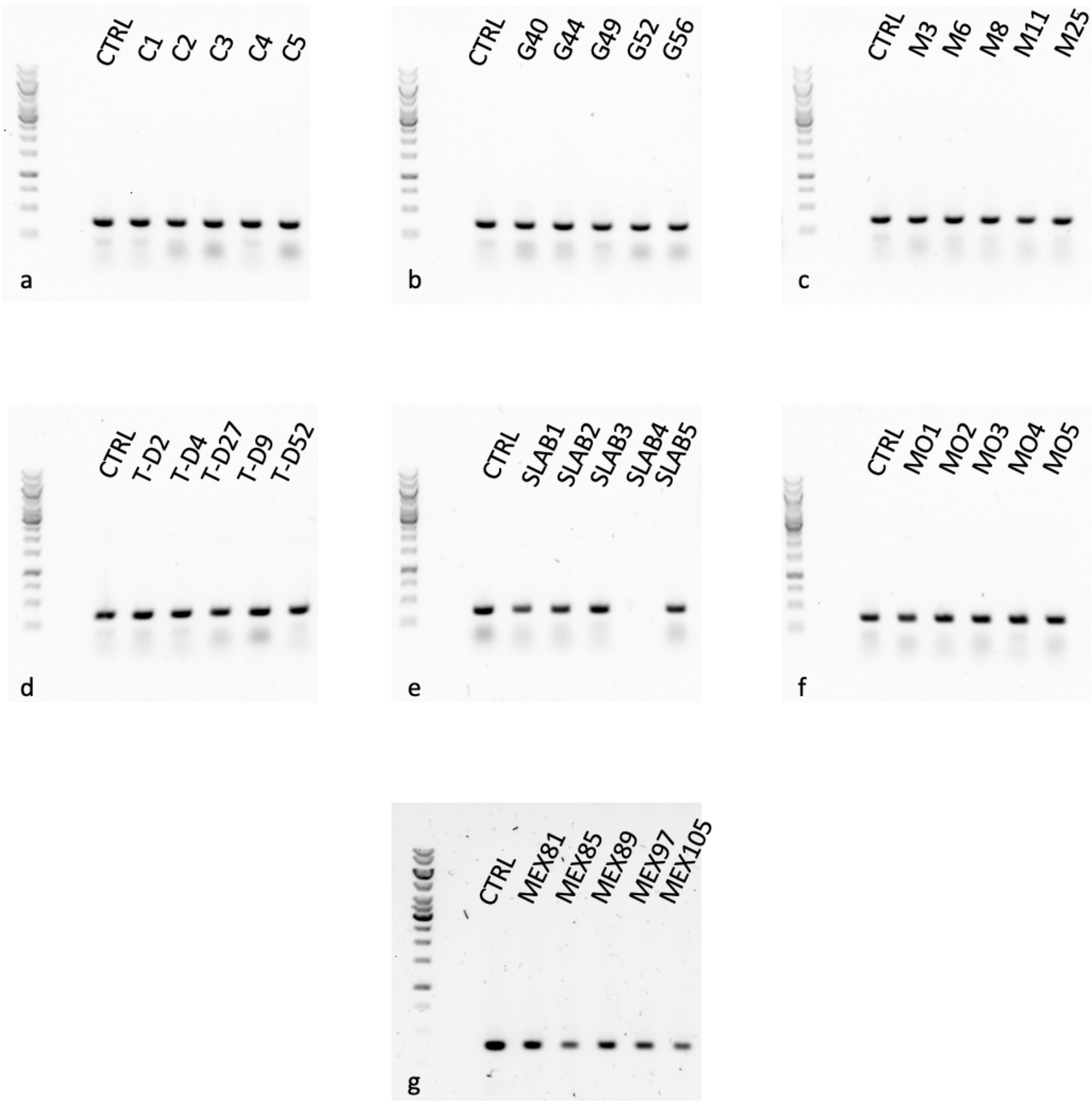

Supplementary Figure 6. A ca. 389 bp PCR product which corresponds to the amplification *DnaB-C* and *RelE-1*, termed as Fragment 6, was detected in most ovary samples collected from different regions. a: Cambodia, b: Guadeloupe, c: Martinique, d: Thailand, e: SLAB, f: Montpellier (molestus) and g: Mexico. (Second lane on the gel corresponds to the negative CTRL sample).

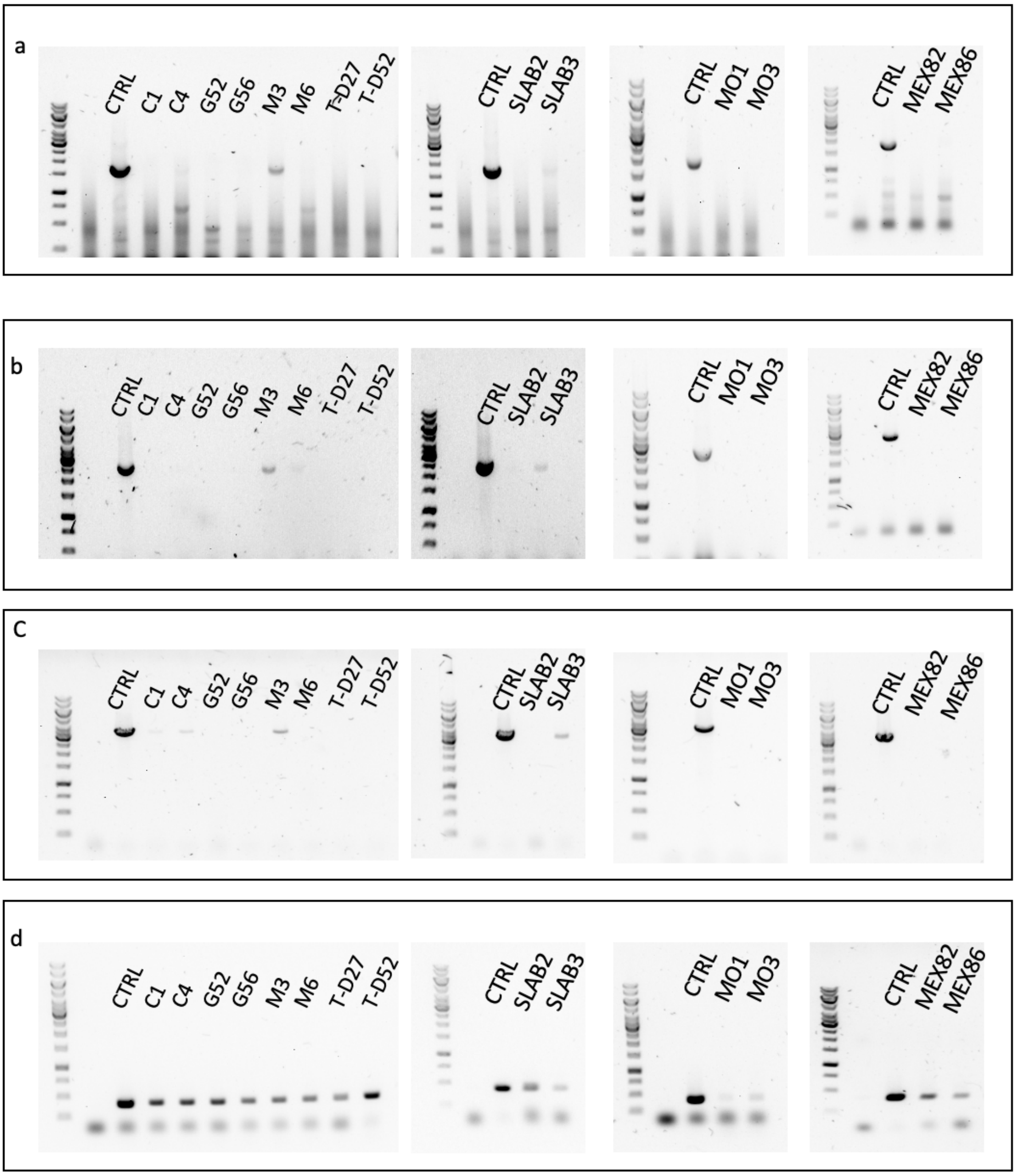

Suppplementary Figure 7. Screening of pWCP in midgut samples. **a**. Amplification of a ca. 1800 bp region that corresponds to *DnaB_C* gene with IS110 (Fragment 1). **b.** Amplification of a ca. 2726 bp (Fragment 3) region that correspond to GP05, GP06 and GP07. **c.** Amplification of a ca. 3451 bp (Fragment 4) region that corresponds to seven genes of the pWCP (GP08, GP09, *ParA*-like, VNTR, GP11, GP12 and *Rel*BE-2). **d.** Amplification of the 16S rRNA gene using specific *Wolbachia* primers. (Second lane on the gel corresponds to the negative CTRL sample).

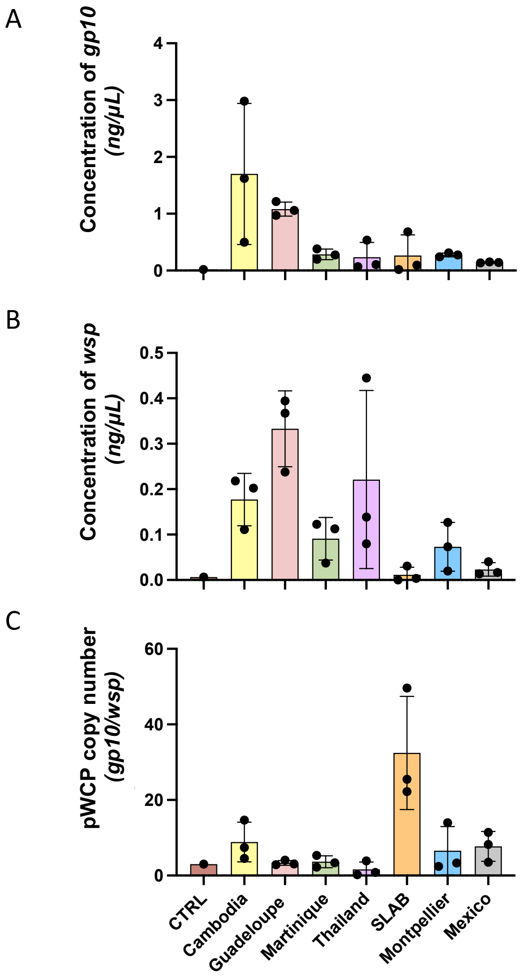

D

Cambodia	Guadeloupe	Martinique	Thailand	SLAB	Montpellier	Mexico	
0,993	>0,9999	>0,9999	>0,9999	0,0308	0,9997	0,9982	CTRL
	0,9652	0,974	0,876	0,0124	0,9998	>0,9999	Cambodia
		>0,9999	>0,9999	0,0021	0,9985	0,9904	Guadeloupe
			>0,9999	0,0023	0,9992	0,9936	Martinique
				0,0012	0,9809	0,9438	Thailand
					0,0058	0,0085	SLAB
						>0,9999	Montpellier

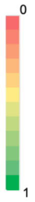

Supplementary Figure 8. Copy number of pWCP in ovary specimens collected from different localities across the globe. A, pWCP concentration as quantified by real-time qPCR using a set of primers targeting the pWCP-borne gene *GP10*. B, *Wolbachia* genome concentration as quantified by real-time qPCR using a set of primers targeting the *wsp* gene. C, pWCP copy number is calculated as the ratio of *GP10* over *wsp* concentrations for each sample. Dots represent the average of three technical replicates for each specimen. Bars show mean values over three specimens analyzed in each locality. Error bars represent the standard deviation from the means. CTRL is the reference *Culex pipiens* sample Cx1, for which the copy number of pWCP was previously estimated at 5 using next generation sequencing approaches [1]. Primer sequences and qPCR conditions are reported in Supplementary Table 4. D, Heat map showing the statistical analysis of the variability of pWCP copy number (panel C) inter-localities. Values represent the p-value from each comparison (red color indicates a p-value <0,05 whereas intermediate and green colors indicate a p-value >0,05).

References:

1. Reveillaud J, Bordenstein SR, Cruaud C, Shaiber A, Esen ÖC, Weill M, et al. Author Correction: The *Wolbachia* mobilome in *Culex pipiens* includes a putative plasmid. *Nat Commun* 2019; **10**: 3153.
